# Mass spectrometry-based proteomics identify novel serum osteoarthritis biomarkers

**DOI:** 10.1186/s13075-022-02801-1

**Published:** 2022-05-23

**Authors:** Ginette Tardif, Frédéric Paré, Clarisse Gotti, Florence Roux-Dalvai, Arnaud Droit, Guangju Zhai, Guang Sun, Hassan Fahmi, Jean-Pierre Pelletier, Johanne Martel-Pelletier

**Affiliations:** 1 Osteoarthritis Research Unit, University of Montreal Hospital Research Centre (CRCHUM), 900 Saint-Denis, Suite R11.412B, Montreal, QC H2X 0A9 Canada; 2 CHU de Québec Research Center, Laval University, Quebec, QC G1V 4G2 Canada; 3 Division of Biomedical Sciences (Genetics), Memorial University of Newfoundland, St. John’s, NL A1B 3V6 Canada; 4 Discipline of Medicine, Memorial University of Newfoundland, St. John’s, NL A1B 3V6 Canada

**Keywords:** Serum biomarkers, Proteomics, Osteoarthritis, Mass spectrometry

## Abstract

**Background:**

Osteoarthritis (OA) is a slowly developing and debilitating disease, and there are no validated specific biomarkers for its early detection. To improve therapeutic approaches, identification of specific molecules/biomarkers enabling early determination of this disease is needed. This study aimed at identifying, with the use of proteomics/mass spectrometry, novel OA-specific serum biomarkers. As obesity is a major risk factor for OA, we discriminated obesity-regulated proteins to target only OA-specific proteins as biomarkers.

**Methods:**

Serum from the Osteoarthritis Initiative cohort was used and divided into 3 groups: controls (*n*=8), OA-obese (*n*=10) and OA-non-obese (*n*=10). Proteins were identified and quantified from the liquid chromatography–tandem mass spectrometry analyses using MaxQuant software. Statistical analysis used the Limma test followed by the Benjamini-Hochberg method. To compare the proteomic profiles, the multivariate unsupervised principal component analysis (PCA) followed by the pairwise comparison was used. To select the most predictive/discriminative features, the supervised linear classification model sparse partial least squares regression discriminant analysis (sPLS-DA) was employed. Validation of three differential proteins was performed with protein-specific assays using plasma from a cohort derived from the Newfoundland Osteoarthritis.

**Results:**

In total, 509 proteins were identified, and 279 proteins were quantified. PCA-pairwise differential comparisons between the 3 groups revealed that 8 proteins were differentially regulated between the OA-obese and/or OA-non-obese with controls. Further experiments using the sPLS-DA revealed two components discriminating OA from controls (component 1, 9 proteins), and OA-obese from OA-non-obese (component 2, 23 proteins). Proteins from component 2 were considered related to obesity. In component 1, compared to controls, 7 proteins were significantly upregulated by both OA groups and 2 by the OA-obese. Among upregulated proteins from both OA groups, some of them alone would not be a suitable choice as specific OA biomarkers due to their rather non-specific role or their strong link to other pathological conditions. Altogether, data revealed that the protein CRTAC1 appears to be a strong OA biomarker candidate. Other potential new biomarker candidates are the proteins FBN1, VDBP, and possibly SERPINF1. Validation experiments revealed statistical differences between controls and OA for FBN1 (*p*=0.044) and VDPB (*p*=0.022), and a trend for SERPINF1 (*p*=0.064).

**Conclusion:**

Our study suggests that 4 proteins, CRTAC1, FBN1, VDBP, and possibly SERPINF1, warrant further investigation as potential new biomarker candidates for the whole OA population.

**Supplementary Information:**

The online version contains supplementary material available at 10.1186/s13075-022-02801-1.

## Introduction

Osteoarthritis (OA), the most common musculoskeletal disorder, is a multifactorial disease irreversibly affecting several joint tissues, the knee being the most prevalent [1]. OA is a major cause of pain, disability, and comorbidities, and about 30% of the worldwide population aged 50 years and older suffer from this disease [2, 3]. OA progression is influenced by numerous factors including age, gender, obesity (major risk factors), and inflammatory mediators, to name a few.

At present, there are no treatments to cure this disease; the current ones only target symptomatic relief. This is related, in part, to the inability to diagnose OA at an early stage, as the existing methods are not sensitive enough. Early and specific OA diagnosis would allow early and targeted treatments/interventions to prevent or delay not only the progression of the disease but also surgery such as joint replacement. This would result in less pain and a better quality of life for patients, in addition to reducing the substantial societal economic burden [4–6].

Because the alteration of the articular tissues develops over a few years, the identification of specific molecules/biomarkers that would enable OA early determination is proving to be a challenging task. To date, there are no regulatory agency-approved biomarkers, as none has yet reached the required specificity, sensitivity, and reliability.

Over the years, several approaches, such as genomics, antibody signature, and metabolomics, have been used to identify biochemical and physiological aspects of OA [7–10]. Another interesting avenue in the search for biomarkers is proteomics. Compared to metabolomics and genomics, the proteomic approach has the advantage of reflecting the patient’s condition at a specific time as well as being more stable than metabolites.

Proteomics using liquid chromatography–tandem mass spectrometry (LC-MS/MS) can identify and quantify thousands of proteins in a single analysis using a relatively small sample amount, which is ideal for the high throughput analysis of a high dynamic range sample such as serum [11]. Such a proteomic approach has been used to identify specific diagnostic markers of many pathologies such as cancer, cardiovascular, liver, and kidney diseases [12–16], as well as some arthritic diseases [8, 17–24], to name a few. LC-MS/MS has been used to monitor the individual proteomes of healthy or OA joint tissues (cartilage, meniscus, synovial membrane), cells (chondrocyte, synoviocyte), and fluids (serum/plasma, synovial fluid, urine) [19, 25–29]. Several proteins that may relate to OA pathological mechanisms have been found but, as mentioned above no molecule has been validated as a specific marker for OA patients, not to mention the early stages of this disease. This could be due in part to the non-specificity of the molecules, which is related more to pathological conditions other than OA, including obesity [30–34].

Therefore, there is an urgent need to identify novel and specific biomarkers that will prove to be both efficient and sensitive enough to be used for OA early diagnosis. The objective of this study was to identify, with the use of LC-MS/MS, novel OA-specific serum biomarkers.

## Material and methods

### Study participants

Participants were selected from the control and progressor subcohorts of the Osteoarthritis Initiative (OAI) database. The individuals in the progressor cohort had symptomatic radiographic OA as described (https://oai.nih.gov). Serum samples were from 8 controls and 20 OA, the latter equally divided into OA-obese (*n*=10; body mass index ≥30 kg/m^2^) and OA-non-obese (*n*=10; BMI <30 kg/m^2^).

For validation purposes, fasting plasma samples were derived from the Newfoundland and Labrador cohort in which the controls were from the Complex Diseases in Newfoundland population: Environment and Genetics (CODING) [35] and the OA samples from the Newfoundland Osteoarthritis Study (NFOAS; https://www.med.mun.ca/NFOAS/Home.aspx) [36]. Plasma samples were from 20 controls and 20 OA, the latter equally divided into OA-obese (*n*=10) and OA-non-obese (*n*=10).

The characteristics of the selected individuals are listed in Table 1 (OAI) and Table 2 (CODING and NFOAS). For the OAI, the demographic, clinical, and radiographic data were obtained from the OAI database (https://oai.nih.gov).

**Table 1:** Osteoarthritis Initiative (OAI) participant characteristics

	Control (*n*=8)	OA (*n*=20)	***p***^**‡**^	OA-obese (*n*=10)	***p***^**‡**^	OA-non-obese (*n*=10)	***p***^**‡**^	***p***^**§**^
Age, years	56 ± 3	65 ± 9	**0.037**	62 ± 9	0.182	67 ± 9	**0.023**	0.441
Gender, male, % (*n*)	25% (2)	60% (12)	0.121	60% (6)	0.188	60% (6)	0.188	1.000
BMI, kg/m^2^	24.7 ± 4.1	30.8 ± 5.4	**0.011**	35.0 ± 4.1	**0.0002**	26.6 ± 2.0	0.286	**0.047**
WOMAC								
Pain (0–20)	0	3.2 ± 2.5	**<0.0001**	2.8 ± 2.8	**0.0003**	3.5 ± 2.3	**0.0003**	0.577
Function (0–68)	0	12.3 ± 9.4	**0.0003**	10.7 ± 10.6	**0.001**	14.0 ± 8.2	**0.0004**	0.644
Stiffness (0–8)	0	2.5 ± 1.2	**<0.0001**	2.2 ± 1.0	**0.0003**	2.7 ± 1.3	**0.0003**	0.487
Total (0–96)	0	17.9 ± 12.0	**<0.0001**	15.7 ± 13.2	**0.0003**	20.2 ± 10.9	**0.0003**	0.552
Kellgren-Lawrence grade, % (*n*)			**<0.0001**		**<0.0001**		**<0.0001**	0.160
0–1	100% (7)*	0% (0)		0% (0)		0% (0)		
2	0% (0)	0% (0)		0% (0)		0% (0)		
3	0% (0)	35% (7)		50% (5)		20% (2)		
4	0% (0)	65% (13)		50% (5)		80% (8)		
Medial joint space width, mm	3.9 ± 0.4 *	1.0 ± 0.8	**<0.0001**	1.2 ± 0.9	**0.001**	0.9 ± 0.8	**0.001**	0.739

Data are presented as mean ± standard deviation (SD) or as indicated. Continuous variables were compared using the Mann-Whitney test. Fisher’s exact test was used for gender, and chi-square test for Kellgren-Lawrence

*p*-values in bold indicate statistical significance. Comparison were *p*^‡^, to control group; *p*^§^, OA-obese and OA-non-obese groups

*BMI*, body mass index; *OA*, osteoarthritis; *WOMAC*, Western Ontario and McMaster Universities Osteoarthritis Index

*One missing value in the control group

**Table 2:** Complex Diseases in Newfoundland population: Environment and Genetics (CODING) (control) and the Newfoundland Osteoarthritis Study (NFOAS) (OA) participant characteristics

	Control (*n*=20)	OA (*n*=20)	***p***^**‡**^
Age, years	63.7 ± 3.6	63.7 ± 3.3	0.903
Gender, male, % (*n*)	50% (10)	50% (10)	1.000
BMI, kg/m^2^	31.1 ± 4.4	30.6 ± 3.8	0.579
WOMAC			
Pain (0–20)	NA	14.6 ± 4.1	-
Function (0–68)	NA	46.9 ± 8.2	-
Stiffness (0–8)	NA	6.1 ± 1.3	-
Total (0–96)	NA	67.5 ± 12.1	-

Data are presented as mean ± standard deviation (SD) or as indicated. Continuous variables were compared using the Mann-Whitney test. Fisher’s exact test was used for gender

*p*-values in bold indicate statistical significance. Comparison were *p*^‡^, to control group

The Kellgren-Lawrence and the joint space width were not done before the surgery

*BMI*, body mass index; *NA*, not available; *OA*, osteoarthritis; *WOMAC*, Western Ontario and McMaster Universities Osteoarthritis Index

All participants had provided written informed consent for their participation. For the OAI cohort, the ethics approval was obtained by each of the OAI clinical sites (University of Maryland Baltimore Institutional Review Board, Ohio State University’s Biomedical Sciences Institutional Review Board, University of Pittsburgh Institutional Review Board, and Memorial Hospital of Rhode Island Institutional Review Board) and the OAI coordinating center (Committee on Human Research at the University of California, San Francisco, CA, USA). For the CODING and NFOAS cohorts, the ethics approval was obtained from the Health Research Ethics Board of Newfoundland and Labrador.

The Institutional Ethics Committee Board of the University of Montreal Hospital Research Centre approved the use of the human serum/plasma.

### Serum/plasma samples

Serum/plasma samples were obtained from the OAI (refer to the OAI operations manual detailing specimen collection and processing methods [https://oai.nih.gov]) and the CODING/NFOAS, as previously described [36, 37]. The specimens were collected after an overnight fast using a uniform protocol. For the plasma, blood was collected and plasma separated from the red cells immediately after collection by centrifugation (20,000 rpm for 10 min). Upon reception, samples for both cohorts were aliquoted, stored frozen at −80°C, and thawed at 4°C just before use.

### Mass spectrometry

#### Preparation of serum samples

Data for the samples (non-depleted and depleted) were both acquired in Data Dependent Acquisition mode and analyzed with MaxQuant software, version 1.6.7 [38], as previously described [39].

The non-depleted samples were randomized before analysis. One microliter of each serum sample was diluted in 24 μl of sodium deoxycholate (SDC) buffer consisting of 1% deoxycholate/10 mM Tris (2-carboxyethyl)phosphine/40 mM chloroacetamide/100 mM Tris pH 8.5, heated for 10 min at 95°C, followed by treatment with a mixture of trypsin and Lys-C (Promega, Madison, WI, USA) (0.66 μg of each enzyme) for 1 h at 37°C. The digestion was stopped with 5 μl 50% formic acid causing the precipitation of deoxycholate. The samples were then centrifuged at 16,000*g* for 15 min at 4°C.

The peptides contained in the supernatant were purified on StageTips C18 Empore (3M, St-Paul, MN, USA) according to Rappsilber et al*.* [40]. Finally, the peptides were vacuum dried and stored at −20°C prior to mass spectrometry analysis.

#### High-abundance protein depletion for building a matching library

To improve the number of peptides/protein identification, in the final analysis, a matching library was prepared for its use with the MaxQuant software, as described by Geyer et al. [41]. This used a depleted serum. By adding a library of depleted serum in the analysis, this strategy took advantage of the “match between runs” function of the MaxQuant software, where peptides identified by MS/MS in the library can be matched to the non-depleted samples to recover their quantification even without MS/MS. This library was obtained by pooling 2 μl of each patient’s serum sample, which was then depleted for high abundance proteins using the Seppro IgY14 Spin Column kit according to the manufacturer protocol (Sigma-Aldrich, St Louis, MO, USA). The flow-through was collected, and the proteins were precipitated with the addition of 5 volumes of ice-cold acetone and incubated overnight at −20°C. After centrifugation at 10,000*g* for 10 min, the pellet was resuspended by 120 μl of SDC buffer and heated at 95°C for 10 min. After cool down, the pooled samples were digested with 1:100 Trypsin:proteins and 1:100 Lys-C:proteins ratios according to a Bradford protein assay. The resulting peptides were purified on Oasis HLB Cartridge (Waters) according to the manufacturer’s procedure. The peptides were then fractionated on a high pH reversed-phase peptide chromatography according to Yang et al. [42]. The 12 resulting fractions were vacuum dried and stored at −20°C prior to mass spectrometry analysis.

#### Liquid chromatography (LC)-MSMS analysis

Both non-depleted samples and fractions of the depleted pool were analyzed, as previously described [43]. In brief, samples or fractions were resuspended with 30 μl 2% acetonitrile/0.05% trifluoroacetic acid. Protein concentration was determined at 205 nm using a NanoDrop 2000 spectrophotometer (Thermo Scientific, Waltham, MA, USA); the protein concentration was adjusted to 0.2 μg/μl. Five microliters of the resuspended peptide digestion (equivalent to 1 μg peptides) was injected on a nanoflow liquid chromatography/MSMS (nanoflow LC-tandem MS). The experiments were performed with a Dionex UltiMate 3000 nanoRSLC chromatography system (Thermo Fisher Scientific/Dionex Softron GmbH, Germering, Germany) connected to an Orbitrap Fusion Tribrid ETD mass spectrometer (Thermo Fisher Scientific, San Jose, CA, USA) equipped with a nano electrospray ion source. Peptides were trapped at 20 μl/min in a loading solvent (2% acetonitrile, 0.05% trifluoroacetic acid [44]) on a 5-mm length 300 μm Internal Diameter (I.D.), 5 μm particles Acclaim™ PepMap™ 100 pre-column cartridge (Thermo Fisher Scientific/Dionex Softron GmbH) for 5 minutes. Then, the pre-column was switched online with 500-mm length, 75 μm I.D., 3 μm particles, Acclaim™ PepMap™ 100 C18 analytical column (Thermo Fisher Scientific/Dionex Softron GmbH), and the peptides were eluted with a linear gradient from 5 to 40% (A: 0,1% formic acid, B: 80% acetonitrile, 0.1% formic acid) for 90 min, at 300 nl/min. Mass spectra were acquired using a Data Dependent Acquisition mode (Thermo XCalibur software, version 4.3). Full scan mass spectra (350 to 1800 m/z) were acquired in the orbitrap using an automatic gain control (AGC) target of 4e5, a maximum injection time of 50 ms, and a resolution of 120,000. Internal calibration using lock mass on the m/z 445.12003 siloxane ion was used. Each MS scan was followed by the acquisition of fragmentation MSMS spectra of the most intense ions for a total cycle time of 3 s (highest speed mode). The selected ions were isolated using the quadrupole analyzer in a window of 1.6 m/z and fragmented by higher energy collision-induced dissociation (HCD) with 35% of collision energy. The resulting fragments were detected by the linear ion trap at a rapid scan rate with an AGC target of 1e4 and a maximum injection time of 50 MS. Dynamic exclusion of previously fragmented peptides was set for a period of 20 s and a tolerance of 10 ppm.

#### Database searching and label-free quantification

Spectra were searched against a human proteins database (Uniprot *Homo sapiens* Reference Proteome – UP000005640 – 74435 entries - 21.04.2019) using the Andromeda module of the MaxQuant software [39]. In brief, the trypsin/P enzyme parameter was selected with two possible missed cleavages. Carbamidomethylation of cysteines was set as a fixed modification, methionine oxidation, and deamidation of glutamine and asparagine as variable modifications. Mass search tolerances were 5 ppm and 0.5 Dalton for MS and MS/MS, respectively. For protein validation, a maximum false discovery rate of 1% at peptide and protein levels was used based on a target/decoy search. MaxQuant was also used for label-free quantification with a minimum ratio count of 1. The “match between runs” algorithm was used with 20 min as alignment time window and 0.7 min as match time window values to enable a peptide MS1 signal match between the matching library consistent with fractions of depleted samples and the non-depleted serum samples. Only unique and razor peptides were used for quantification. All other parameters were set at default values.

#### Protein assays

Proteins tested were the fibrillin-1 (FBN1), Vitamin D-binding protein (VDBP), and SERPINF1. They were determined with specific assays according to manufacturer’s specifications. FBN1 was quantitated by ELISA (dilution 1:5; #MBS3804755, MyBiosource, San Diego, CA, USA), VDBP with a Multiplex assay (dilution 1:10000; #HCCBP2MAG-58K, EMD Millipore Corporation, Billerica, MA, USA), and SERPINF1, by Luminex assay (dilution 1:4000; #LXSAHM-01, R&D systems, Minneapolis, MN, USA). Protein quantification was performed using the LiquiChip 200 apparatus, and the data analysis performed with ht LiquiChip Analyzer software (Qiagen, Toronto, ON, Canada). For each biomarker, an 8-point standard curve and appropriate controls were included, and samples were done in duplicate. The minimum detectable doses were for FBN1, 0.312 ng/ml; VDBP, 0.58 ng/ml; and SERPINF1, 3.66 pg/ml.

#### Data treatment and statistical analysis

The proteinGroups.txt file generated by MaxQuant was used in R software, version 3.4 [45]. The intensity values of each peptide in each non-depleted serum sample were normalized using the median of all intensity values in each sample (normalization by column). For each comparison, only peptides having at least 60% of non-missing values across all the non-depleted samples were considered as quantifiable. Missing values remaining after this filtering were imputed using a noise value calculated as the first centile of all intensity values per sample (calculation per column), as previously described [46]. Only proteins with at least two quantified peptides were kept for further analysis.

For the analysis of differential expression between two groups, a protein ratio was calculated using the average of protein intensities in all samples of the same group. These ratios were then converted into *z-*score (*z* = (*x-μ*)/*σ* where *x* =log_2_(ratio); *μ* = average of all log_2_(ratios); *σ* =standard deviation of all log_2_(ratios)) for data centering. Statistical analysis was performed using the Limma Bioconductor package [47] to define the probability of variation (*p*-value) of each protein between two groups. This method has been preferred to the usual Student *t*-test as it has been shown to be less sensitive to the number of biological replicates. This was followed by the Benjamini-Hochberg method to adjust for multiple comparison (*q*-value). Proteins with a *q*-value < 0.050 and absolute value of *z*-score > 1.96 were considered significantly different.

Further, two multivariate methods were used through the MixOmics R package [48]. First, to compare the proteomic profiles, the multivariate unsupervised principal component analysis (PCA) [49] followed by the pairwise comparison were used. PCA method enables to cluster the samples by reducing the dimension of expression data with minimum information loss and visualize the similarities between the proteins. It is a logistic regression that provide a relative weighting of the protein importance. Second, to select the most predictive/discriminative features, the supervised classification model sparse partial least squares regression discriminant analysis (sPLS-DA) [50] was used. This method is a linear classification model enabling discriminative variable selection that could predict the outcome. It allows to seek for components that best separate the samples. Moreover, this method presented a graphical representation of the components and proteins assisting for the interpretation of the results. The number of components and variables was defined after a tuning step to optimize the distinction between the three groups (control, OA-obese, OA-non-obese).

For the validation experiments, the differences between groups were assessed using the Student *t*-test. A value of *p*≤0.050 was considered statistically significant. Statistical analysis was performed using the GraphPad Prism 8 (San Diego, CA, USA).

## Results

### Subject characteristics

Table 1 shows the characteristics of the participants from the OAI cohort comparing control, OA, OA-obese, and OA-non-obese individuals. The obese/non-obese division was performed in an attempt to discriminate proteins not specific to OA but to obesity. Compared to controls, OA patients were older (*p*=0.037) and had higher BMI (*p*=0.011), Western Ontario and McMaster Universities Osteoarthritis Index (WOMAC) scores (*p*≤0.0003), Kellgren-Lawrence grades (*p*<0.0001), and smaller medial joint space width (*p*<0.0001). Comparison between OA-obese with OA-non-obese showed only, and as expected, that the former had a higher BMI (*p*=0.047). When each two OA subgroups were compared to control, data were comparable to the total OA group, but OA-non-obese were slightly older (*p*=0.023) and had a similar BMI.

Table 2 shows that none of the participant characteristics differed between the CODING (controls) and NFOAS (OA) cohorts. Compared to the OAI controls, the CODING participants were older (*p*<0.0001) and had a higher BMI (*p*=0.002), and OA participants from the NFOAS had higher WOMAC scores (*p*<0.0001) than those from the OAI.

### Quantitative proteomic analysis

#### Principal component analysis (PCA)

**Fig. 1:**
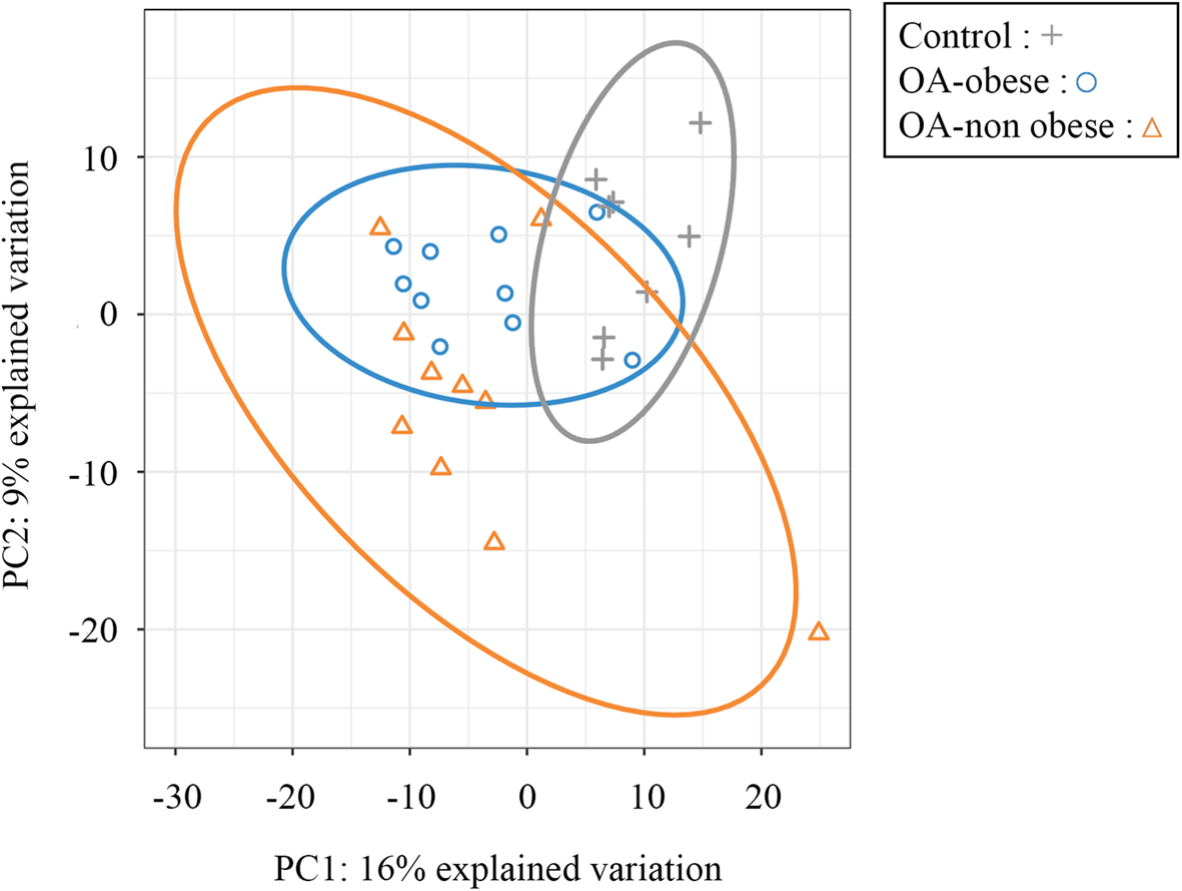
Principal component analysis (PCA) (unsupervised). Serum samples were from controls (*n*=8; + [gray]), osteoarthritis (OA)-obese (*n*=10; ○ [blue]), and OA-non obese (*n*=10; Δ [orange]) and analyzed by PCA. PCA represents the maximum variability that exists between different samples and unsupervised means regardless of the group to which they belong. The closer the points are in the PCA, the closer the proteomic profiles of the corresponding samples. In each axis, the percentage represents the total variability between all points. PC, principal component

A shotgun proteomic analysis was performed on the non-depleted individual serum samples. Five hundred and nine (509) proteins could be identified in at least one individual sample. As mentioned above, in addition to the non-depleted, we added a depleted serum library for the database searching and quantification. Such an addition boosted the protein identification by 28%. Two hundred and seventy-nine (279) proteins (Table S1) were quantified after filtering for proteins having at least 60% of non-missing values in at least one of the two compared conditions and having two quantified peptides or more to retain only high-quality protein measurements. This data was used to explore the global proteomic profile of each sample and group through a PCA analysis. This unsupervised multivariate method (Fig. 1) generates principal component axes that best explain the variability in the data without knowing the group of the sample. The data showed that the three groups (control, OA-obese, OA-non-obese) could not be clearly distinguished based on their global proteomic profile suggesting that the differences between the groups might be from low variations in protein expression and/or variations on a small number of protein species.

#### Pairwise differential expression analysis

To unveil the small differences between the groups, pairwise differential analyses were performed using the protein quantitative value. Comparisons were made between control with OA-non-obese and OA-obese as well as between OA-obese with OA-non-obese. For each comparison, protein ratios were calculated between the two groups and converted into *z*-score for data centering. Statistical analysis was performed with the Limma method. Table S2 lists the normalized intensity values, means, ratios, and *z*-scores for the 12 proteins that were found significantly differentially expressed in at least one of the three pairwise comparisons, and Table 3 summarizes the data. Of note, differential expressions could not be performed for FBN1, comparing OA-obese with controls, and for lysine-specific demethylase4C/4E/4B (KDM4C/4B/4E), comparing OA-non-obese with controls, as these proteins could not be quantitated accurately due to missing values in OA-obese and OA-non-obese groups, respectively (Tables S2 and Table 3).

**Table 3:** Principal component analysis-pairwise differential expression

	OA-obese/control		OA-non-obese/control	
Protein (gene designation)	z-score/log_**2**_(ratio)/***q***-value	Regulation	***z***-score/log_**2**_(ratio)/***q***-value	Regulation
C-reactive protein (CRP)	**4.21/3.47/ 0.05**	**↑**	**3.35/ 2.72/ 0.04**	**↑**
Lysozyme C (LYZ)	**3.94/3.29/0.0002**	**↑**	**4.45/ 3.40/ 0.03**	**↑**
Cartilage acidic protein 1 (CRTAC1)	**2.47/ 2.27/ 0.01**	**↑**	**3.19/ 2.62/ 0.003**	**↑**
Prostaglandin-H2 D-isomerase (PTGDS)	**2.57/ 2.34/0.0002**	**↑**	**2.57/ 2.23/*0.054***	**↑**
Immunoglobulin delta chain C region (IGHD)	**5.91/ 4.65/0.04**	**↑**	1.28/ 1.43/ 0.74	**-**
Lysine-specific demethylase4C/4E/4B (KDM4C/4B/4E)	**2.01/ 1.96/ 0.01**	**↑**	NA	
KH-Type Splicing Regulatory Protein (KHSRP)	2.07/ 2.00/ 0.10	**-**	**2.80/2.37/0.04**	**↑**
Immunoglobulin heavy variable 3-35 (IGHV3-35)	1.71/ 1.75/0.34	**-**	**3.15/ 2.59/ 0.02**	**↑**
Protein S100A9 (S100A9)	1.13/ 1.34/ 0.17	**-**	**2.05/ 1.91/ 0.02**	**↑**
Fibrillin-1 (FBN1)	NA		**2.99/ 2.49/ 0.02**	**↑**
Adiponectin (ADIPOQ)	**-3.57/ -1.91/ 0.04**	**↓**	-2.73/-1.06/ 0.17	**-**
Actins (ACTA1/ACTC1/ACTG2/ACTA2)	**-3.83/ -2.08/0.04**	**↓**	-2.00/ -0.61/ 0.16	**-**

Serum samples were obtained from control (*n*=8), osteoarthritis (OA)-obese (*n*=10), and OA-non-obese (*n*=10) individuals, prepared for mass spectrometry (MS) and analyzed by principal component analysis (PCA). The intensity values for each protein and for each sample were obtained from the MS analysis output, and the mean of the intensity values was calculated for each protein (for the protein, refer to Table S2). The ratio of the means intensity values was used to calculate the *z*-score and Limma *q*-value for each protein. A protein was considered upregulated if the *z*-score was >1.96, the log_2_(ratio) >0 and the Limma *q*-value <0.05; inversely, a protein was considered downregulated with a *z*-score <1.96, a log_2_(ratio) <0 and a Limma *q*-value <0.05. The values of statistically differentially regulated proteins are indicated in bold and the value in italic indicates a strong trend towards statistical difference. Of note, all the proteins reported in this table have MS/MS sequences in addition to being in the library

- indicates proteins with no differential regulation; NA (not applicable) refers to proteins for which there were too many missing values to assign a final score

**Fig. 2:**
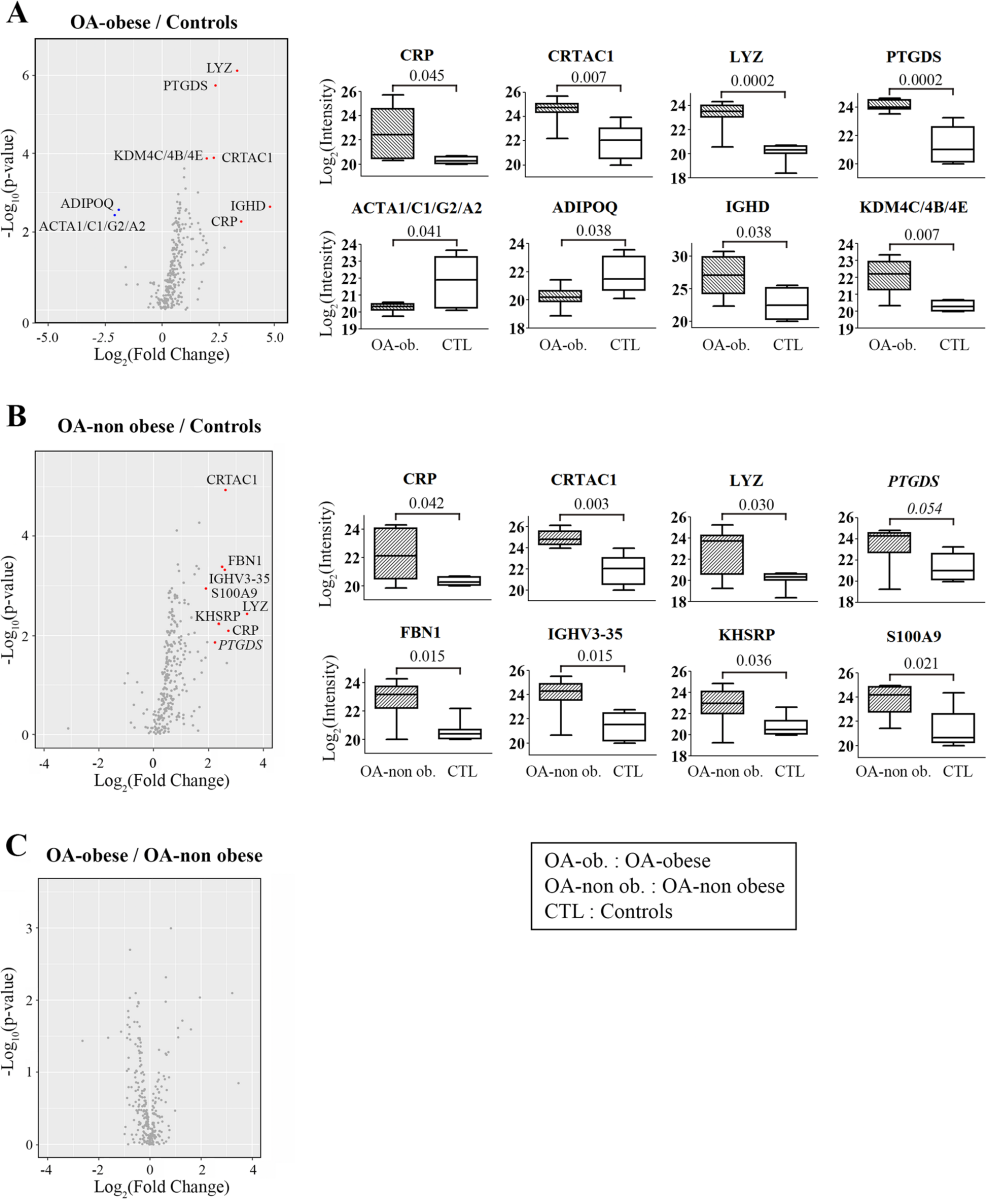
Principal component analysis (PCA)-pairwise differential expression. Volcano and box plots of statistically differently regulated proteins (for the volcano plot, red when osteoarthritis (OA) was lower than controls and blue when OA was higher than controls between **A** OA-obese (*n*=10, OA-ob. [hatched]) and controls (*n*=8, CTL [white]), **B** OA-non obese (*n*=10, OA-non ob. [hatched]) and controls (*n*=8, CTL [white]) and **C** OA-obese (*n*=10) and OA-non obese (*n*=10)). The intensity values for each protein and sample were obtained from the mass spectrometry analysis (refer to Table S2) and transformed as Log_2_. Statistical analysis used the Limma method, and *q*<0.050 was considered statistically significant; a strong trend toward statistical difference is indicated in italic. ACT, actins; ADIPOQ, adiponectin; CRP, C-reactive protein; CRTAC1, cartilage acidic protein 1; FBN, fibrillin 1; IGHV3-35, Ig heavy variable 3-35; KDM4C/4B/4E, lysine-specific demethylase4C/4E/4B; KHSRP, KH-Type Splicing Regulatory Protein; LYZ, lysozyme; PTGDS, prostaglandin-H2 D-isomerase; S100A9: S100 Calcium Binding Protein A9

For these 12 proteins, pairwise comparison revealed that 8 were differentially regulated between OA-obese with controls, and also 8 between OA-non-obese with controls; some proteins being common to both comparisons (Table 3, Fig. 2A, B). No protein was found differentially regulated between the two OA subgroups (Fig. 2C). One may also note that, in Fig. 2A, B, the ratio distribution is not centered when OA obese and OA non-obese are compared to control. In the latter, there are slightly less quantified proteins; however, the overall intensity is somewhat strong. Although this cannot be explained at present, to overcome this issue, we centered the data by calculating a *z*-score and considered proteins as regulated or not between two conditions based on both their q-value and z-score.

Compared to controls, data revealed that in the OA-obese group (Fig. 2A, Table 3), CRP, CRTAC1, LYZ, PTGDS, IGHD, and KDM4C/4B/4E were all upregulated, whereas ACTA1/ACTC1/ACTG2/ACTA2 and ADIPOQ were downregulated. In the OA-non-obese/control comparison (Fig. 2B), CRP, CRTAC1, LYZ, PTGDS, FBN1, IGHV3-35, KHSRP, and S100A9 were all upregulated in the OA-non-obese; PTGDS was included in the upregulated proteins as the *q*-value (*q*=0.054) showed a strong trend towards significance.

#### Sparse partial least squares regression discriminant analysis (sPLS-DA)

The pairwise comparison between the two OA subgroups did not reveal proteins that were significantly different and that could be related to the obesity condition. To mine deeper into the data and unveil proteins related to obesity, not specific necessarily to OA, we performed another multivariate analysis, the sPLS-DA. This supervised analysis enabled the selection of the most discriminative proteins in the data to classify the samples [50].

**Fig. 3:**
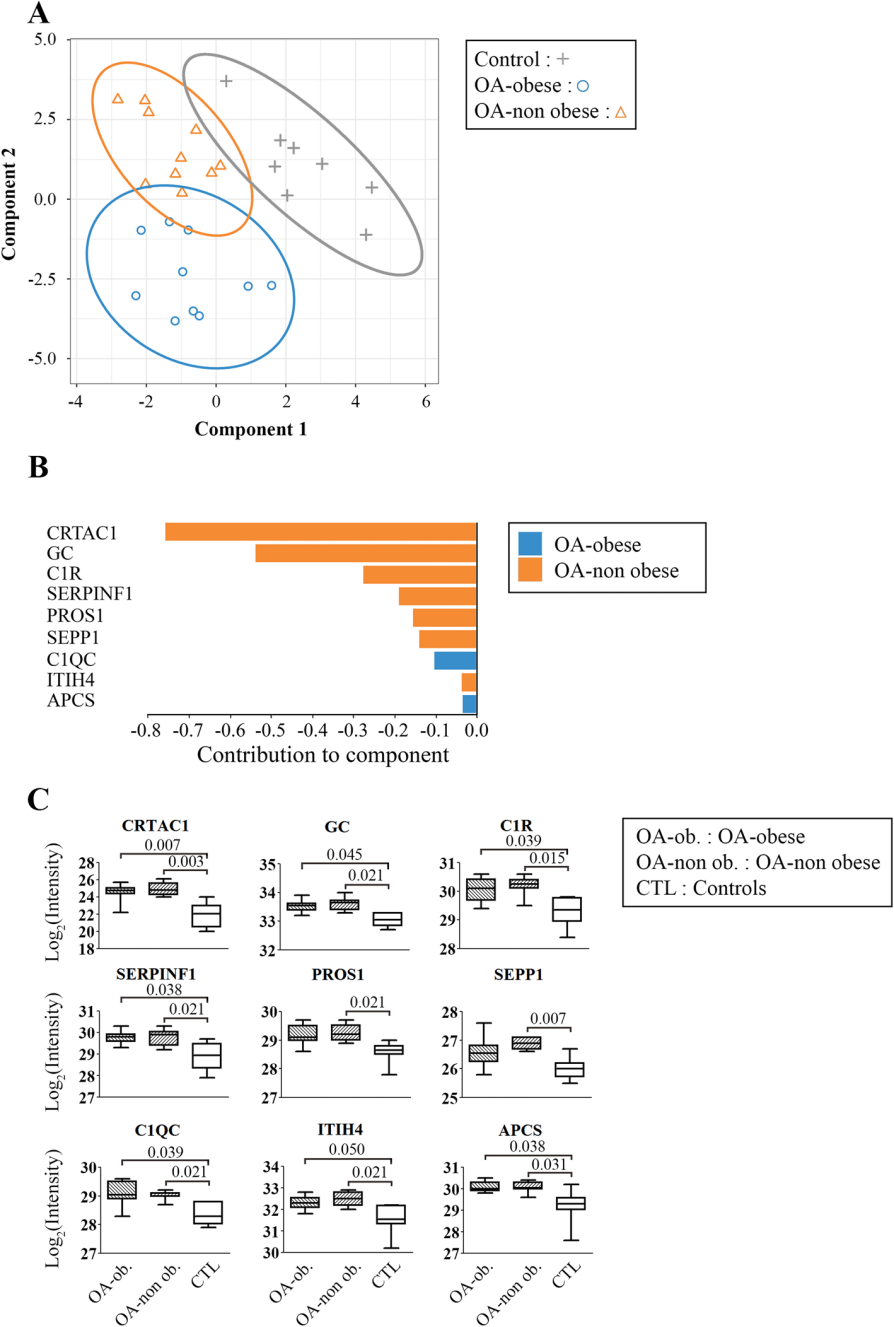
Sparse partial least squares discriminant analysis (sPLS-DA) contribution to component 1. Serum samples were from controls (*n*=8), osteoarthritis (OA)-obese (*n*=10) and OA-non obese *n*=10) and analyzed by sPLS-DA. **A** The dot plot component 1 vs. component 2 allowed for the identification of component 1 discriminating both the OA-obese (o [blue]) and OA-non obese (Δ [orange]) groups from controls (+ [gray]). **B** Contribution of the 9 proteins in component 1; the plots display the loading weight and indicate the class (OA-obese [blue]; OA-non obese,[orange]) for which the selected protein has a maximal mean value; the negative value indicates contributions higher in the OA compared to the control group. **C** Box plots of each protein comprised in component 1. The intensity values for each protein and each sample were obtained from the mass spectrometry analysis, and the mean of the intensity values was calculated for each protein and transformed as Log_2_. Statistical analysis used the Limma method, and *q*<0.050 was considered statistically different. OA-obese (OA-ob. [hatched left]), OA-non obese (OA-non ob. [hatched right], and control (CTL [white]). CRTAC1, cartilage acidic protein 1; GC, vitamin D binding protein; C1R, complement C1r subcomponent; Serpin F1, pigment epithelium-derived factor; PROS1, vitamin K-dependent protein S; SEPP1, selenoprotein P; C1QC, complement C1q subcomponent subunit C; ITIH4, inter-alpha-trypsin inhibitor heavy chain 4; APCS, serum amyloid P

**Fig. 4:**
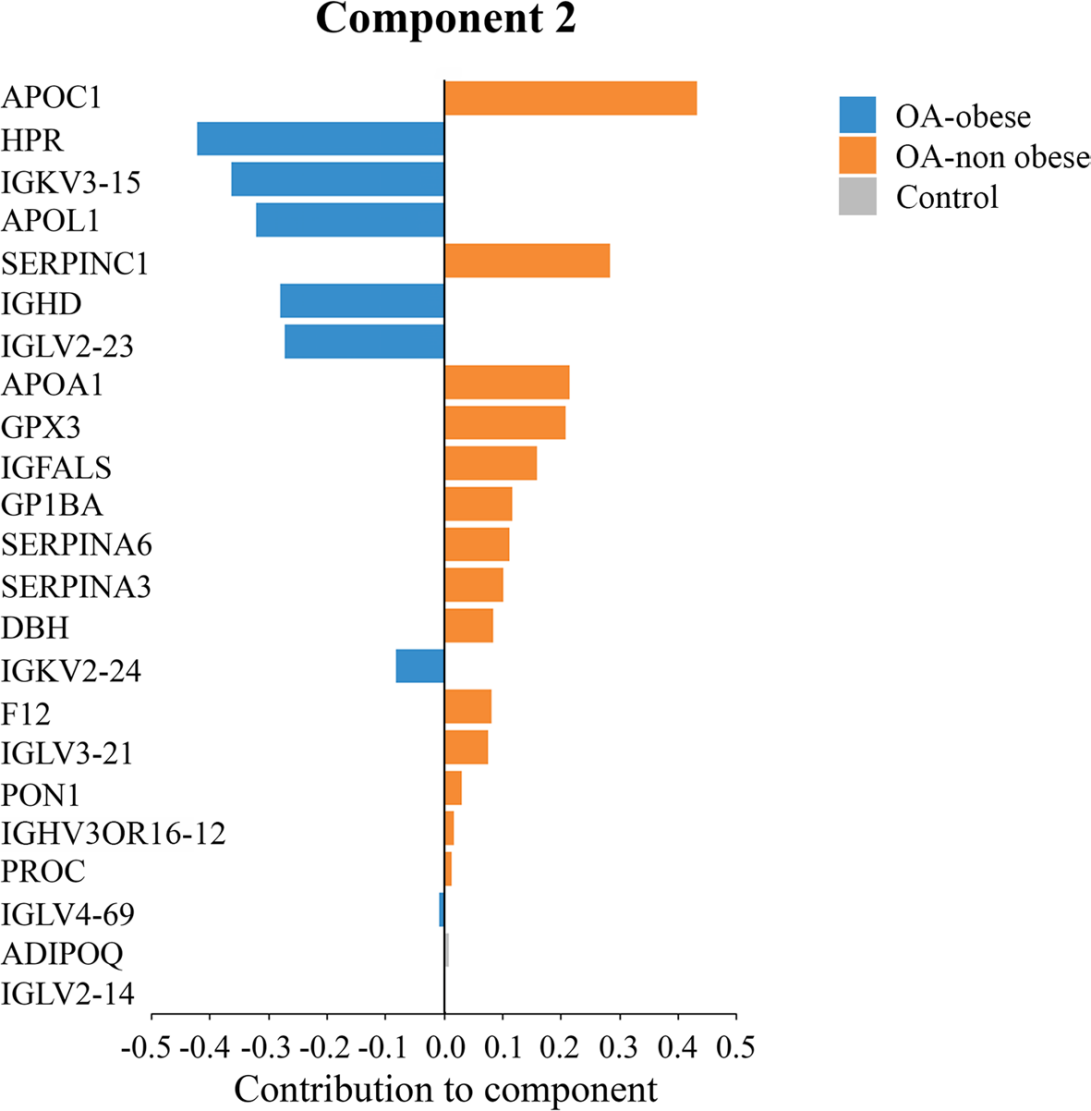
Sparse partial least squares discriminant analysis (sPLS-DA) contribution to component 2. Serum samples were obtained from osteoarthritis (OA)-obese (*n*=10) and OA-non obese (*n*=10) individuals and analyzed by sPLS-DA. Contribution of the 23 proteins in component 2; the plots display the loading weight and indicate the class (OA-obese, [blue]; OA-non obese, [orange]; control, [gray]) for which the selected protein has a maximal mean value. The negative value indicates contributions higher in the OA-obese compared to the OA-non obese group, and positive number higher values in the OA-non obese. ADIPOQ, adiponectin; APOA1, apolipoprotein A-I; APOC1, apolipoprotein C-I; APOL1, apolipoprotein L1; DBH, dopamine beta hydroxylase; F12, coagulation factor XII; GP1BA, platelet glycoprotein Ib alpha chain; GPX3, glutathione peroxidase 3; HPR, haptoglobin-related protein; IGHD, Ig delta chain C region; IGFALS, insulin-like growth factor-binding protein complex acid labile subunit; IGLV2-14, Ig lambda chain V-II region TOG; IGLV4-69, Ig lambda variable 4-69; IGKV2-24, Ig kappa variable 2-24; IGLV2-23, Ig lambda chain V-II region NEI; IGKV3-15, Ig kappa chain V-III region POM; IGLV3-21, Ig lambda chain V-III region LOI; IGHV3OR16-12, Ig Heavy Variable 3/OR16-12 (Non-Functional); PON1, serum paraoxonase/arylesterase 1; PROC, vitamin K-dependent protein C; SERPINA6, corticosteroid-binding globulin; SERPINA3, alpha-1-antichymotrypsin; SERPINC1, antithrombin-III

Data revealed that a very good classification (area under the curve [AUC] >95%) was obtained with two components. Component 1 (9 proteins; Fig. 3) comprised proteins discriminating the two OA groups from the controls, and component 2 (23 proteins; Fig. 4) discriminated the OA-non-obese from the OA-obese. In a given component, each protein does contribute in combination but not equally to the discrimination process, i.e., when a protein is removed from a component, the discriminatory strength of the component is altered.

Figure 3A illustrates a clear separation of the control group from the two OA subgroups, which is particularly visible in component 1. Figure 3B shows the contribution of each of the 9 proteins comprised in component 1 listed by order of importance—CRTAC1, GC, C1R, SERPINF1, PROS1, SEPP1, C1QC, ITIH4, and APCS. Of note, CRTAC1, which was found to contribute the most, was also identified previously in the pairwise analysis as upregulated in both OA-obese and OA-non-obese compared to controls (Fig. 2, Table 3). Figure 3C shows the intensities of the 9 proteins contributing to component 1 for each group and their comparisons between the groups. Compared to controls, both OA groups were upregulated for all 9 proteins and statistical difference was reached for all in the OA-non-obese. Although values of both OA-obese and OA-non-obese were relatively similar for all the 9 proteins, comparison between the OA-obese with controls showed that the proteins PROS1 and SEPP1 did not reach statistical difference.

Component 2 is a group of 23 proteins that discriminates OA-obese from OA-non-obese. Figure 4 shows the contribution value of each of these proteins. Importantly, none of the 23 proteins found in component 1, which discriminates OA from controls, and only the protein ADIPOQ (with a very low contribution) were previously identified in pairwise comparison as down-regulated in OA-obese compared to controls (Fig. 2A, Table 3 and Table S2).

Some of the proteins of component 2 were involved in the coagulation/fibrinolysis pathways or lipid metabolism. Also listed are some immunoglobulins, mostly light chains (lambda and kappa variable). Regarding the contribution of each protein to component 2, ApoC1 and SERPINC1 were the proteins with the strongest contribution in the OA-non-obese group, while HPR, IGKV3-15, and APOL1 led in the OA-obese group.

#### Protein validation

To complement this work, comparison of three proteins (FBN1, VDPB and SERPINF1) using plasma from another cohort (CODING and NFOAS) was performed between controls and OA. Data showed that statistical difference was reached when OA was compared to controls for FBN1 (*p*=0.044), and VDPB (*p*=0.022), and a trend toward significance for SERPINF1 (*p*=0.064) (Fig.S1). Of note, no difference was obtained when OA-obese and OA-non-obese were compared for all the three proteins studied (*p*=0.656, *p*=0.104, and *p*=0.315, respectively) (Fig. S1), suggesting that these proteins are not likely obesity-regulated.

## Discussion

The search for a reliable biomarker in OA is an active field of investigation. Our study identified the proteins CRTAC1, FBN-1, VDBP, and possibly SERPINF1 as potential new and OA-specific serum biomarkers.

To gather the most information about the proteomic analysis performed on our serum samples, we first assessed their proteomic profile through an unsupervised PCA analysis, then two methodologies were used to recover the most discriminative proteins involved in each group: a pairwise differential analysis based on the Limma (Student derived) statistical test and a supervised sPLS-DA analysis. The latter enabled us to find proteins discriminating OA-obese from OA-non-obese groups, which was not possible with the pairwise comparisons.

The PCA data revealed that controls can be partially discriminated from OA patients based on their global proteomic profile, while OA-obese and OA-non-obese patients cannot be differentiated. This was confirmed by pairwise differential expression analyses, which revealed that CRP, LYZ, CRTAC1, and PTGDS were all upregulated in OA individuals compared to controls. These proteins are not likely obesity-regulated as they were significantly higher than the controls in both OA-obese and OA-non-obese in addition to not being found differently regulated in the sPLS-DA component 2, which evaluates proteins between the two OA groups.

CRTAC1 appears to be a strong OA biomarker candidate as it is the only protein identified in both pairwise (increased intensity levels in OA compared to controls) and sPLS-DA (highest contribution in component 1) analyses. However, very little is known about this protein and its role, not only in OA but also in normal human physiology. Two splice variants of this gene have been reported and, in regard to articular tissues, the CRTAC1-A being the predominant form in cartilage [51]. In OA knees, studies have reported that it is a glycosylated extracellular molecule found in the inter-territorial matrix of the deep zone of the cartilage as well as in synovial fluid and serum [21, 26, 51]. It is upregulated in late-stage OA cartilage compared to healthy or early OA cartilage [52, 53]. While preparing the present work, a proteomic study done on an Icelandic population (*n*=39,155 including 12,178 OA) corroborates our finding that CRTAC1 was the most strongly associated (among 4792 proteins studied) to OA diagnosis and progression to joint replacement [54]. It asserts that CRTAC1 is a strong and promising biomarker candidate for OA.

FBN1 is an extracellular matrix protein that assembles into microfibrils to form the template for elastic fiber formation. In the pairwise analysis, data showed its upregulation in OA-non-obese compared to controls. In this analysis, unfortunately this protein could not be assessed in the OA-obese as it had too many missing values to assign a final score. However, in the sPLS-DA component 2, this protein did not discriminate OA-non-obese and OA-obese. Complementary experiments confirm the statistical difference of this protein between OA with controls, in addition no difference was found between OA-non-obese and OA-obese, thus not likely regulated by obesity factors. FBN1 was previously identified in the synovial fluids of OA patients, but no comparison with controls was done [55]. There are three isoforms of FBN, FBN1 being the most abundant in adult tissues [56]. Related to OA, FBN1 has been reported to sequester a key factor involved in the disease’s cartilage and bone, the latent TGF-β1 complex, regulating its bioavailability [57–59]. In addition, FBN1 was found associated with two other musculoskeletal diseases, systemic sclerosis and Marfan syndrome [60–63]. FBN1 would be an interesting molecule for further analysis as a potential OA biomarker.

Other proteins were found upregulated in OA compared to controls, CRP, LYZ, and PTGDS. However, they alone would not be suitable choices as specific OA biomarkers due to their rather non-specific role (CRP, a general marker of inflammation [64, 65]; LYZ, an antibacterial role or their strong link to other pathological conditions (PTGDS) [66–70]. Nonetheless, it is worth mentioning that a ratio of serum CRP with another molecule (monocyte chemoattractant protein-1 [MCP-1]) was suggested as an OA biomarker. This ratio has been found associated with OA symptoms and predicted, in combination with other factors, OA individuals with knee structural degenerative progression [37, 71]. Furthermore, CRP is also known to activate the classical complement pathway by binding to C1q [72]. Although we did not identify C1q in the PCA-pairwise analysis, it was found as a contributor to component 1 in the sPLS-DA analysis.

Several other proteins showed differential regulation in pairwise analysis, but are likely obesity-related, and thus not specific to all OA population. These included IGHD and KDM4C, which were upregulated in OA-obese, and KHSRP, S100A9, and IGHV3-35 were so in OA-non obese, whereas ADIPOQ and ACT were downregulated only in OA-obese.

The sPLS-DA complemented the differential expression findings and further identified proteins that discriminated both OA-obese and OA-non-obese from controls (component 1), as well as OA-obese from OA-non-obese individuals (component 2). This analysis offers an insight into which proteins contribute and how important the contribution of each is towards the discrimination of given groups.

Several proteins comprising component 1 (OA vs. controls) are molecules for which there are few or no reports as to their association with OA, as such offering novel potential candidates for OA biomarker research. The sPLS-DA revealed that the abovementioned CRTAC1 protein contributed the most towards the discrimination of OA and controls. The second contributor being VDBP and validation experiments demonstrated a significant difference between OA and controls and, as for FBN1, not between the OA subgroups. This is a multifunctional protein that not only binds to vitamin D but also has several other different physiological functions such as actin scavenging, binding of fatty acids, and chemotaxis [73]. There has been only one OA study showing increased levels of VDBP and vitamin D receptors in muscles from patients with end-stage knee OA compared to controls [74]. As knee muscles are gaining great interest regarding their impact on OA progression, this protein should be studied further as an OA biomarker.

Two other proteins in component 1, C1R and C1QC, are directly involved in the first step of the classical complement cascade. Of note, the contribution of C1QC is from the OA-obese individuals, thus probably related to obesity. C1 proteases can also cleave non-complement proteins including the LDL receptor-related protein 6, IGFBP5, and nucleolin [75]. The presence of complement proteins in this list was not unexpected, as previous studies reported the activation of the complement cascade in OA [37, 76, 77]. As complement proteins are activated in various diseases as well as in general inflammation processes, the abovementioned proteins would therefore not be very useful as specific OA markers. It has previously been reported that one of the complement proteins, as for the CRP, when employed in ratio with another molecule could be of use as a biomarker for OA cartilage degradation in OA-obese individuals. Hence, the adipokine adipsin, a component of the alternative complement pathway, when combined as a ratio with MCP-1 was found strongly associated with knee cartilage volume loss in OA-obese individuals [37].

SERPINF1, as its name indicates, belongs to the serpin family, but does not display the serine protease inhibitory activity shown by many of its family members. The SERPINF1 gene codes for the pigment epithelium-derived factor (PEDF), which was found to exacerbate mice joint cartilage damage in an in vivo inflammatory joint destruction model (monosodium iodoacetate) [78]. However, PEDF production in the joint is somewhat controversial as it was found upregulated in human OA cartilage in two studies [78, 79], while another showed no expression in articular chondrocytes but an up-regulation in osteophytic chondrocytes [80]. In regard to a musculoskeletal disease, the heritable disorder osteogenesis imperfecta, characterized by bone fragility and low bone mass, is caused by mutations in the SERPINF1 gene [81, 82]. Validation experiments showed that there was a numerical trend toward significance when OA was compared to controls. However, this protein needs more support as a potential OA biomarker and further analysis is suggested.

The other less-contributing proteins in the sPLS-DA component 1 included PROS, a vitamin K-dependent plasma protein that functions as a cofactor for the anticoagulant protease (activated protein C) in the degradation of coagulation factors Va and VIIIa; SEPP1, a selenoprotein implicated as an extracellular antioxidant, and in the transport of selenium to extra-hepatic tissues; ITIH4, a member of the serine protease inhibitor family with diverse functions such as a matrix-stabilizing molecule [83]; and APCS (amyloid P component serum), a glycoprotein capable of binding to apoptotic cells at an early stage and associated with the innate immune system. As for the C1QC, the contribution of APCS in component 1 is from the OA-obese individuals, thus probably related to obesity. Although all these proteins were not specifically studied with respect to their role in OA, some have been associated with other arthritis pathologies including rheumatoid arthritis [84, 85], lupus [86], Kashin-Beck [87], and ankylosing spondylitis [88].

Data from sPLS-DA’s component 2 offered important information related to differentially regulated proteins between OA-obese and OA-non-obese, which are potentially related to obesity. Obesity is a well-known and major risk factor for OA, but not all OA patients are obese. Thus, in the search for a specific OA biomarker, it is important to focus on molecules that are regulated in the general OA population, avoiding other pathological condition-related (obesity) proteins. Among the 23 proteins identified in component 2, none were found in component 1 (discriminating OA from controls), and thus are mostly related to conditions other than OA. Several of these proteins are involved in lipid metabolism: apolipoproteins A1, C1, and L1, paraoxonase 1 (PON1), which binds to HDL, HPR, which is known to associate with APOL1-containing HDL, and ADIPOQ, an adipokine involved in the control of fat metabolism and insulin sensitivity, which is also listed in the PCA analysis. Notably, a number of apolipoproteins and serpins identified as part of component 2 are among the highest contributors, and a number of those proteins have been studied as to their presence/levels in OA [19, 89–94]. Some others, such as SERPINC1, coagulation factor XII (involved in contact activation pathways), and protein C (PROC), are involved in the coagulation/fibrinolysis pathways, which are known to be activated in OA, as well as in obesity [95–100]. However, it cannot be ascertained that these pathways are specific of OA or rather of obesity. It is our opinion that the use of those proteins in the search for specific OA biomarkers should not be pursued. Nevertheless, in the OA-obese subgroup, some of these proteins including APOA1 and SERPINC1 would be worthwhile studying, as they may amplify/accelerate the OA process in these people and thus be used as therapeutic targets.

Although our study has identified potential biomarkers, it has limitations. First, the cohort used (proteomic, OAI; validation CODING and NFOAS) included individuals from the USA and Canada, respectively. A validation of our results from other countries would be required to determine whether those proteins could indeed be further studied as biomarkers. Second, gender discrimination could also be performed as it is well known that there are sex-specific differences in OA [101–103]. In this study, we could not perform such a discrimination as we had a relatively modest sample size, which was limited by the methodology used. A technique allowing a greater sample size should permit it. Third, despite a data filtering, some of the proteins (for example CRP, KDM4C, FBN1, and actin) across the whole dataset showed a high number of imputed noise values (Table S1), which might have created a bias in the reported fold changes. However, as some of the targeted proteins selected as new potential biomarkers for the entire OA population were further validated using samples from an external cohort, including FBN1, this reduces the risk to report wrong biomarkers. Moreover, the use of a larger cohort combined with other proteomic analysis strategies could confirm our findings.

## Conclusion

In OA, current diagnoses are not sensitive enough to identify the disease in the early stages. To improve therapeutic approaches for the prevention or delay of the progression of this disease, the identification of specific molecules/biomarkers enabling early determination of this disease is needed. At present, there are no such validated specific serum biochemical markers. As a novel contribution, we identified, by using proteomics/mass spectrometry and targeted disease-specific proteins, four OA serum potential new biomarker candidates for the entire OA population: CRTAC1, FBN1, VDBP, and possibly SERPINF1.

## Supplementary Information

**Additional file 1: Table S1**. Mass Spectrometry Protein Identification. **Table S2**. Pairwise differential expression analysis.

**Additional file 2: Figure S1**. Protein validation in plasma.

## Data Availability

Data from the Osteoarthritis Initiative (OAI) cohort are publicly available (https://data-archive.nimh.nih.gov/oai/). All mass spectrometry data (raw files and MaxQuant search result files) are publicly available on ProteomeXchange repository (www.proteomexchange.org) with the identifier PXD032112. Additional data may be obtained upon a reasonable request to JMP, as long as the request is evaluated as scientifically relevant and pertinent.

## Abbreviations

ACT: Actins
ADIPOQ: Adiponectin
A0A0G2JMB2: Ig alpha-2 chain C region (fragment)
APCS: Serum amyloid P
APOA1: Apolipoprotein A-I
APOC1: Apolipoprotein C-I
APOL1: Apolipoprotein L1
C1QC: Complement C1q subcomponent subunit C
C1R: Complement C1r subcomponent
CFHR4: Complement factor H-related protein 4
CRP: C-reactive protein
CRTAC1: Cartilage acidic protein 1
DBH: Dopamine beta-hydroxylase
FBN1: Fibrillin 1
FETUB: Fetuin-B
F12: Coagulation factor XII
GC_(VDBP): Vitamin D-binding protein
GP1BA: Platelet glycoprotein Ib alpha chain;Glycocalicin
GPX3: Glutathione peroxidase;Glutathione peroxidase 3
HPR: Haptoglobin-related protein
Ig: Immunoglobulin
IGHD: Ig delta chain C region
IGFALS: Insulin-like growth factor-binding protein complex acid labile subunit
IGLV2-14: Ig lambda chain V-II region TOG
IGLV4-69: Ig lambda variable 4-69
IGKV2-24: Ig kappa variable 2-24
IGLV2-23: Ig lambda chain V-II region NEI
IGKV3-15: Ig kappa chain V-III region POM
IGLV3-21: Ig lambda chain V-III region LOI
IGHV3-35: Ig heavy variable 3-35
IGHV3OR16-12: Ig Heavy Variable 3/OR16-12 (Non-Functional)
ITIH4: Inter-Alpha-Trypsin Inhibitor Heavy Chain 4
KDM4C/4B/4E: Lysine-specific demethylase4C/4E/4B
KHSRP: KH-Type Splicing Regulatory Protein
LYZ: Lysozyme
MCP-1: Monocyte chemoattractant protein-1
MS: Mass spectrometry
OA: Osteoarthritis
PCA: Principal component analysis
PON1: Serum paraoxonase/arylesterase 1
PROC: Vitamin K-dependent protein C
PROS1: Vitamin K-dependent protein S
PTGDS: Prostaglandin-H2 D-isomerase
SEPP1: Selenoprotein P
SERPINA6: Corticosteroid-binding globulin
SERPINA3: Alpha-1-antichymotrypsin
SERPINC1: Antithrombin-III
SERPINF1: Pigment epithelium-derived factor
sPLS-DA: Sparse partial least squares regression discriminant analysis
S100A9: S100 Calcium Binding Protein A9

## References

[1] Felson DT, Naimark A, Anderson J, Kazis L, Castelli W, Meenan RF (1987). The prevalence of knee osteoarthritis in the elderly. The Framingham Osteoarthritis Study. Arthritis Rheum.

[2] Cross M, Smith E, Hoy D, Nolte S, Ackerman I, Fransen M (2014). The global burden of hip and knee osteoarthritis: estimates from the global burden of disease 2010 study. Ann Rheum Dis..

[3] Yoshimura N, Muraki S, Nakamura K, Tanaka S (2017). Epidemiology of the locomotive syndrome: The research on osteoarthritis/osteoporosis against disability study 2005-2015. Mod Rheumatol..

[4] Neogi T (2013). The epidemiology and impact of pain in osteoarthritis. Osteoarthritis Cartilage..

[5] Kingsbury SR, Gross HJ, Isherwood G, Conaghan PG (2014). Osteoarthritis in Europe: impact on health status, work productivity and use of pharmacotherapies in five European countries. Rheumatology (Oxford)..

[6] Xie F, Kovic B, Jin X, He X, Wang M, Silvestre C (2016). Economic and humanistic burden of osteoarthritis: a systematic review of large sample studies. Pharmacoeconomics..

[7] Heard BJ, Rosvold JM, Fritzler MJ, El-Gabalawy H, Wiley JP, Krawetz RJ (2014). A computational method to differentiate normal individuals, osteoarthritis and rheumatoid arthritis patients using serum biomarkers. J R Soc Interface..

[8] Lourido L, Ayoglu B, Fernandez-Tajes J, Oreiro N, Henjes F, Hellstrom C (2017). Discovery of circulating proteins associated to knee radiographic osteoarthritis. Sci Rep..

[9] Camacho-Encina M, Balboa-Barreiro V, Rego-Perez I, Picchi F, VanDuin J, Qiu J (2019). Discovery of an autoantibody signature for the early diagnosis of knee osteoarthritis: data from the Osteoarthritis Initiative. Ann Rheum Dis..

[10] Carlson AK, Rawle RA, Wallace CW, Brooks EG, Adams E, Greenwood MC (2019). Characterization of synovial fluid metabolomic phenotypes of cartilage morphological changes associated with osteoarthritis. Osteoarthritis Cartilage..

[11] Gharbi M, Deberg M, Henrotin Y (2011). Application for proteomic techniques in studying osteoarthritis: a review. Front Physiol..

[12] Saleem S, Tariq S, Aleem I, Sadr-Ul S, Tahseen M, Atiq A (2019). Proteomics analysis of colon cancer progression. Clin Proteomics..

[13] Borne Y, Fagerberg B, Sallsten G, Hedblad B, Persson M, Melander O (2019). Biomarkers of blood cadmium and incidence of cardiovascular events in non-smokers: results from a population-based proteomics study. Clin Proteomics..

[14] Niu L, Geyer PE, Wewer Albrechtsen NJ, Gluud LL, Santos A, Doll S (2019). Plasma proteome profiling discovers novel proteins associated with non-alcoholic fatty liver disease. Mol Syst Biol..

[15] Pena MJ, Mischak H, Heerspink HJ (2016). Proteomics for prediction of disease progression and response to therapy in diabetic kidney disease. Diabetologia..

[16] Hanash S (2011). Progress in mining the human proteome for disease applications. OMICS..

[17] Gobezie R, Kho A, Krastins B, Sarracino DA, Thornhill TS, Chase M (2007). High abundance synovial fluid proteome: distinct profiles in health and osteoarthritis. Arthritis Res Ther..

[18] Fischer R, Trudgian DC, Wright C, Thomas G, Bradbury LA, Brown MA (2012). Discovery of candidate serum proteomic and metabolomic biomarkers in ankylosing spondylitis. Mol Cell Proteomics..

[19] Takinami Y, Yoshimatsu S, Uchiumi T, Toyosaki-Maeda T, Morita A, Ishihara T (2013). Identification of potential prognostic markers for knee osteoarthritis by serum proteomic analysis. Biomark Insights..

[20] Wanner J, Subbaiah R, Skomorovska-Prokvolit Y, Shishani Y, Boilard E, Mohan S (2013). Proteomic profiling and functional characterization of early and late shoulder osteoarthritis. Arthritis Res Ther..

[21] Ritter SY, Collins J, Krastins B, Sarracino D, Lopez M, Losina E (2014). Mass spectrometry assays of plasma biomarkers to predict radiographic progression of knee osteoarthritis. Arthritis Res Ther..

[22] Sierra-Sanchez A, Garrido-Martin D, Lourido L, Gonzalez-Gonzalez M, Diez P, Ruiz-Romero C (2017). Screening and validation of novel biomarkers in osteoarticular pathologies by comprehensive combination of protein aarray technologies. J Proteome Res..

[23] Malekzadeh A, Leurs C, van Wieringen W, Steenwijk MD, Schoonheim MM, Amann M (2019). Plasma proteome in multiple sclerosis disease progression. Ann Clin Transl Neurol..

[24] Mun S, Lee J, Park A, Kim HJ, Lee YJ, Son H, et al. Proteomics approach for the discovery of rheumatoid arthritis biomarkers using mass spectrometry. Int J Mol Sci. 2019;20(18):4368. 10.3390/ijms20184368 PMC6769564 31491989

[25] Fernandez-Puente P, Mateos J, Fernandez-Costa C, Oreiro N, Fernandez-Lopez C, Ruiz-Romero C (2011). Identification of a panel of novel serum osteoarthritis biomarkers. J Proteome Res..

[26] Ritter SY, Subbaiah R, Bebek G, Crish J, Scanzello CR, Krastins B (2013). Proteomic analysis of synovial fluid from the osteoarthritic knee: comparison with transcriptome analyses of joint tissues. Arthritis Rheum..

[27] Steinberg J, Ritchie GRS, Roumeliotis TI, Jayasuriya RL, Clark MJ, Brooks RA (2017). Integrative epigenomics, transcriptomics and proteomics of patient chondrocytes reveal genes and pathways involved in osteoarthritis. Sci Rep..

[28] Hsueh MF, Khabut A, Kjellstrom S, Onnerfjord P, Kraus VB (2016). Elucidating the molecular composition of cartilage by proteomics. J Proteome Res..

[29] Folkesson E, Turkiewicz A, Englund M, Onnerfjord P (2018). Differential protein expression in human knee articular cartilage and medial meniscus using two different proteomic methods: a pilot analysis. BMC Musculoskelet Disord..

[30] Coggon D, Reading I, Croft P, McLaren M, Barrett D, Cooper C (2001). Knee osteoarthritis and obesity. Int J Obes Relat Metab Disord..

[31] Johnson VL, Hunter DJ (2014). The epidemiology of osteoarthritis. Best Pract Res Clin Rheumatol..

[32] Thijssen E, van Caam A, van der Kraan PM (2015). Obesity and osteoarthritis, more than just wear and tear: pivotal roles for inflamed adipose tissue and dyslipidaemia in obesity-induced osteoarthritis. Rheumatology (Oxford)..

[33] Berenbaum F, Wallace IJ, Lieberman DE, Felson DT (2018). Modern-day environmental factors in the pathogenesis of osteoarthritis. Nat Rev Rheumatol..

[34] Misra D, Fielding RA, Felson DT, Niu J, Brown C, Nevitt M (2019). Risk of knee osteoarthritis with obesity, sarcopenic obesity, and sarcopenia. Arthritis Rheumatol..

[35] Fontaine-Bisson B, Thorburn J, Gregory A, Zhang H, Sun G (2014). Melanin-concentrating hormone receptor 1 polymorphisms are associated with components of energy balance in the Complex Diseases in the Newfoundland Population: Environment and Genetics (CODING) study. Am J Clin Nutr..

[36] Werdyani S, Liu M, Zhang H, Sun G, Furey A, Randell EW (2021). Endotypes of primary osteoarthritis identified by plasma metabolomics analysis. Rheumatology (Oxford)..

[37] Martel-Pelletier J, Tardif G, Rousseau Trepanier J, Abram F, Dorais M, Raynauld JP (2019). The ratio adipsin/MCP-1 is strongly associated with structural changes and CRP/MCP-1 with symptoms in obese knee osteoarthritis subjects: data from the Osteoarthritis Initiative. Osteoarthritis Cartilage..

[38] Cox J, Hein MY, Luber CA, Paron I, Nagaraj N, Mann M (2014). Accurate proteome-wide label-free quantification by delayed normalization and maximal peptide ratio extraction, termed MaxLFQ. Mol Cell Proteomics..

[39] Sheta R, Roux-Dalvai F, Woo CM, Fournier F, Bourassa S, Bertozzi CR (2016). Proteomic dataset for altered glycoprotein expression upon GALNT3 knockdown in ovarian cancer cells. Data Brief..

[40] Rappsilber J, Mann M, Ishihama Y (2007). Protocol for micro-purification, enrichment, pre-fractionation and storage of peptides for proteomics using StageTips. Nat Protoc..

[41] Geyer PE, Kulak NA, Pichler G, Holdt LM, Teupser D, Mann M (2016). Plasma proteome profiling to assess human health and disease. Cell Syst..

[42] Yang F, Shen Y, Camp DG, Smith RD (2012). High-pH reversed-phase chromatography with fraction concatenation for 2D proteomic analysis. Expert Rev Proteomics..

[43] Sheta R, Woo CM, Roux-Dalvai F, Fournier F, Bourassa S, Droit A (2016). A metabolic labeling approach for glycoproteomic analysis reveals altered glycoprotein expression upon GALNT3 knockdown in ovarian cancer cells. J Proteomics..

[44] Adamczyk L, Adkins JK, Agakishiev G, Aggarwal MM, Ahammed Z, Alekseev I (2014). Beam-energy dependence of the directed flow of protons, antiprotons, and pions in Au+Au collisions. Phys Rev Lett..

[45] R Core Team. R: a language and environment for statistical computing. Vienna: R Foundation for Statistical Computing; 2017. https://www.R-project.org/.

[46] Lazar C, Gatto L, Ferro M, Bruley C, Burger T (2016). Accounting for the multiple natures of missing values in label-free quantitative proteomics data sets to compare imputation strategies. J Proteome Res..

[47] Ritchie ME, Phipson B, Wu D, Hu Y, Law CW, Shi W (2015). limma powers differential expression analyses for RNA-sequencing and microarray studies. Nucleic Acids Res..

[48] Rohart F, Gautier B, Singh A, Cao K-AL (2017). mixOmics: An R package for ‘omics feature selection and multiple data integration. PLoS Comput Biol..

[49] Jolliffe IT, Cadima J (2016). Principal component analysis: a review and recent developments. Philos Trans Ser A Math Phys Eng Sci..

[50] Le Cao KA, Boitard S, Besse P (2011). Sparse PLS discriminant analysis: biologically relevant feature selection and graphical displays for multiclass problems. BMC Bioinformatics..

[51] Steck E, Braun J, Pelttari K, Kadel S, Kalbacher H, Richter W (2007). Chondrocyte secreted CRTAC1: a glycosylated extracellular matrix molecule of human articular cartilage. Matrix Biol..

[52] Ijiri K, Zerbini LF, Peng H, Otu HH, Tsuchimochi K, Otero M (2008). Differential expression of GADD45beta in normal and osteoarthritic cartilage: potential role in homeostasis of articular chondrocytes. Arthritis Rheum..

[53] Aigner T, Fundel K, Saas J, Gebhard PM, Haag J, Weiss T (2006). Large-scale gene expression profiling reveals major pathogenetic pathways of cartilage degeneration in osteoarthritis. Arthritis Rheum..

[54] Styrkarsdottir U, Lund SH, Saevarsdottir S, Magnusson MI, Gunnarsdottir K, Norddahl GL, et al. The CRTAC1 protein in plasma associates with osteoarthritis and predicts progression to joint replacements: a large-scale proteomics scan in Iceland. Arthritis Rheumatol. 2021;73(11):2025–34. 10.1002/art.41793 PMC8596997 33982893

[55] Balakrishnan L, Nirujogi RS, Ahmad S, Bhattacharjee M, Manda SS, Renuse S (2014). Proteomic analysis of human osteoarthritis synovial fluid. Clin Proteomics..

[56] Thomson J, Singh M, Eckersley A, Cain SA, Sherratt MJ, Baldock C (2019). Fibrillin microfibrils and elastic fibre proteins: functional interactions and extracellular regulation of growth factors. Semin Cell Dev Biol..

[57] Chaudhry SS, Cain SA, Morgan A, Dallas SL, Shuttleworth CA, Kielty CM (2007). Fibrillin-1 regulates the bioavailability of TGFbeta1. J Cell Biol..

[58] Nistala H, Lee-Arteaga S, Smaldone S, Siciliano G, Carta L, Ono RN (2010). Fibrillin-1 and -2 differentially modulate endogenous TGF-beta and BMP bioavailability during bone formation. J Cell Biol..

[59] Sengle G, Tsutsui K, Keene DR, Tufa SF, Carlson EJ, Charbonneau NL (2012). Microenvironmental regulation by fibrillin-1. PLoS Genet..

[60] Lee B, Godfrey M, Vitale E, Hori H, Mattei MG, Sarfarazi M (1991). Linkage of Marfan syndrome and a phenotypically related disorder to two different fibrillin genes. Nature..

[61] Ramirez F, Pereira L, Zhang H, Lee B (1993). The fibrillin-Marfan syndrome connection. BioEssays..

[62] Tan FK, Arnett FC, Antohi S, Saito S, Mirarchi A, Spiera H (1999). Autoantibodies to the extracellular matrix microfibrillar protein, fibrillin-1, in patients with scleroderma and other connective tissue diseases. J Immunol..

[63] Villano M, Borghini A, Manetti M, Gabbrielli E, Rossi A, Sestini P (2013). Systemic sclerosis sera affect fibrillin-1 deposition by dermal blood microvascular endothelial cells: therapeutic implications of cyclophosphamide. Arthritis Res Ther..

[64] Bray C, Bell LN, Liang H, Haykal R, Kaiksow F, Mazza JJ (2016). Erythrocyte sedimentation rate and C-reactive protein measurements and their relevance in clinical medicine. WMJ..

[65] Soeki T, Sata M (2016). Inflammatory biomarkers and atherosclerosis. Int Heart J..

[66] Harrington MG, Fonteh AN, Biringer RG, AF RH, Cowan RP. (2006). Prostaglandin D synthase isoforms from cerebrospinal fluid vary with brain pathology. Dis Markers..

[67] Cheung CL, Cheung TT, Lam KS, Cheung BM (2013). Reduced serum beta-trace protein is associated with metabolic syndrome. Atherosclerosis..

[68] White CA, Ghazan-Shahi S, Adams MA (2015). beta-Trace protein: a marker of GFR and other biological pathways. Am J Kidney Dis..

[69] Alves MR, Do Amaral NS, Marchi FA, Silva FIB, Da Costa A, Carvalho KC (2019). Prostaglandin D2 expression is prognostic in highgrade serous ovarian cancer. Oncol Rep..

[70] Choi DJ, An J, Jou I, Park SM, Joe EH (2019). A Parkinson's disease gene, DJ-1, regulates anti-inflammatory roles of astrocytes through prostaglandin D2 synthase expression. Neurobiol Dis..

[71] Bonakdari H, Jamshidi A, Pelletier JP, Abram F, Tardif G, Martel-Pelletier J (2021). A warning machine learning algorithm for early knee osteoarthritis structural progressor patient screening. Ther Adv Musculoskel Dis..

[72] Haapasalo K, Meri S (2019). Regulation of the complement system by pentraxins. Front Immunol..

[73] Delanghe JR, Speeckaert R, Speeckaert MM (2015). Behind the scenes of vitamin D binding protein: more than vitamin D binding. Best Pract Res Clin Endocrinol Metab..

[74] Brennan-Speranza TC, Mor D, Mason RS, Bartlett JR, Duque G, Levinger I (2017). Skeletal muscle vitamin D in patients with end stage osteoarthritis of the knee. J Steroid Biochem Mol Biol..

[75] Lu J, Kishore U (2017). C1 complex: an adaptable proteolytic module for complement and non-complement functions. Front Immunol..

[76] Struglics A, Okroj M, Sward P, Frobell R, Saxne T, Lohmander LS (2016). The complement system is activated in synovial fluid from subjects with knee injury and from patients with osteoarthritis. Arthritis Res Ther..

[77] Wang Q, Rozelle AL, Lepus CM, Scanzello CR, Song JJ, Larsen DM (2011). Identification of a central role for complement in osteoarthritis. Nat Med..

[78] Nakamura DS, Hollander JM, Uchimura T, Nielsen HC, Zeng L (2017). Pigment Epithelium-Derived Factor (PEDF) mediates cartilage matrix loss in an age-dependent manner under inflammatory conditions. BMC Musculoskelet Disord..

[79] Pfander D, Grimmer C, Aigner T, Swoboda B, Schmidt R, Cramer T (2006). Pigment epithelium derived factor--the product of the EPC-1 gene--is expressed by articular chondrocytes and up regulated in osteoarthritis. Ann Rheum Dis..

[80] Klinger P, Beyer C, Ekici AB, Carl HD, Schett G, Swoboda B (2013). The transient chondrocyte phenotype in human osteophytic cartilage: a role of pigment epithelium-derived factor?. Cartilage..

[81] Becker J, Semler O, Gilissen C, Li Y, Bolz HJ, Giunta C (2011). Exome sequencing identifies truncating mutations in human SERPINF1 in autosomal-recessive osteogenesis imperfecta. Am J Hum Genet..

[82] Homan EP, Rauch F, Grafe I, Lietman C, Doll JA, Dawson B (2011). Mutations in SERPINF1 cause osteogenesis imperfecta type VI. J Bone Miner Res..

[83] Zhuo L, Hascall VC, Kimata K (2004). Inter-alpha-trypsin inhibitor, a covalent protein-glycosaminoglycan-protein complex. J Biol Chem..

[84] Kawaguchi H, Matsumoto I, Osada A, Kurata I, Ebe H, Tanaka Y (2018). Identification of novel biomarker as citrullinated inter-alpha-trypsin inhibitor heavy chain 4, specifically increased in sera with experimental and rheumatoid arthritis. Arthritis Res Ther..

[85] Pagani S, Bellan M, Mauro D, Castello LM, Avanzi GC, Lewis MJ (2020). New insights into the role of Tyro3, Axl, and Mer receptors in rheumatoid arthritis. Dis Markers..

[86] Recarte-Pelz P, Tassies D, Espinosa G, Hurtado B, Sala N, Cervera R (2013). Vitamin K-dependent proteins GAS6 and Protein S and TAM receptors in patients of systemic lupus erythematosus: correlation with common genetic variants and disease activity. Arthritis Res Ther..

[87] Sun W, Wang X, Zou X, Song R, Du X, Hu J (2010). Selenoprotein P gene r25191g/a polymorphism and quantification of selenoprotein P mRNA level in patients with Kashin-Beck disease. Br J Nutr..

[88] Lee JH, Jung JH, Kim J, Baek WK, Rhee J, Kim TH (2020). Proteomic analysis of human synovial fluid reveals potential diagnostic biomarkers for ankylosing spondylitis. Clin Proteomics..

[89] Sanchez-Enriquez S, Torres-Carrillo NM, Vazquez-Del Mercado M, Salgado-Goytia L, Rangel-Villalobos H, Munoz-Valle JF (2008). Increase levels of apo-A1 and apo B are associated in knee osteoarthritis: lack of association with VEGF-460 T/C and +405 C/G polymorphisms. Rheumatol Int..

[90] Oliviero F, Sfriso P, Baldo G, Dayer JM, Giunco S, Scanu A (2009). Apolipoprotein A-I and cholesterol in synovial fluid of patients with rheumatoid arthritis, psoriatic arthritis and osteoarthritis. Clin Exp Rheumatol..

[91] Lu M, Lu Q, Zhang Y, Tian G (2011). ApoB/apoA1 is an effective predictor of coronary heart disease risk in overweight and obesity. J Biomed Res..

[92] Ruan X, Li Z, Zhang Y, Yang L, Pan Y, Wang Z (2011). Apolipoprotein A-I possesses an anti-obesity effect associated with increase of energy expenditure and up-regulation of UCP1 in brown fat. J Cell Mol Med..

[93] de Seny D, Cobraiville G, Charlier E, Neuville S, Lutteri L, Le Goff C (2015). Apolipoprotein-A1 as a damage-associated molecular patterns protein in osteoarthritis: ex vivo and in vitro pro-inflammatory properties. PLoS One..

[94] Yanagisawa A, Ueda M, Sueyoshi T, Nakamura E, Tasaki M, Suenaga G (2016). Knee osteoarthritis associated with different kinds of amyloid deposits and the impact of aging on type of amyloid. Amyloid..

[95] Ghosh P, Cheras PA (2001). Vascular mechanisms in osteoarthritis. Best Pract Res Clin Rheumatol..

[96] So AK, Varisco PA, Kemkes-Matthes B, Herkenne-Morard C, Chobaz-Peclat V, Gerster JC (2003). Arthritis is linked to local and systemic activation of coagulation and fibrinolysis pathways. J Thromb Haemost..

[97] Kaye SM, Pietilainen KH, Kotronen A, Joutsi-Korhonen L, Kaprio J, Yki-Jarvinen H (2012). Obesity-related derangements of coagulation and fibrinolysis: a study of obesity-discordant monozygotic twin pairs. Obesity..

[98] Blokhin IO, Lentz SR (2013). Mechanisms of thrombosis in obesity. Curr Opin Hematol..

[99] Samad F, Ruf W (2013). Inflammation, obesity, and thrombosis. Blood..

[100] Vilahur G, Ben-Aicha S, Badimon L (2017). New insights into the role of adipose tissue in thrombosis. Cardiovasc Res..

[101] Boyan BD, Hart DA, Enoka RM, Nicolella DP, Resnick E, Berkley KJ (2013). Hormonal modulation of connective tissue homeostasis and sex differences in risk for osteoarthritis of the knee. Biol Sex Differ..

[102] Boyan BD, Tosi LL, Coutts RD, Enoka RM, Hart DA, Nicolella DP (2013). Addressing the gaps: sex differences in osteoarthritis of the knee. Biol Sex Differ..

[103] Pan Q, O'Connor MI, Coutts RD, Hyzy SL, Olivares-Navarrete R, Schwartz Z (2016). Characterization of osteoarthritic human knees indicates potential sex differences. Biol Sex Differ..

