# Supplementary material for: Mass spectrometry-based proteomics identify novel serum osteoarthritis biomarkers
Source: Arthritis Res Ther. 2022 May 23;24:120. doi: 10.1186/s13075-022-02801-1 (PMC9125906; doi:10.1186/s13075-022-02801-1)

**Manuscript ARRT-D-21-00723.R2**

**Mass spectrometry-based proteomics identify novel serum osteoarthritis biomarkers**

Ginette Tardif, Frédéric Paré, Clarisse Gotti, Florence Roux-Dalvai, Arnaud Droit, Guangju Zhai^c^, Guang Sun, Hassan Fahmi, Jean-Pierre Pelletier, Johanne Martel-Pelletier

**Supplementary Figure**

Figure S1


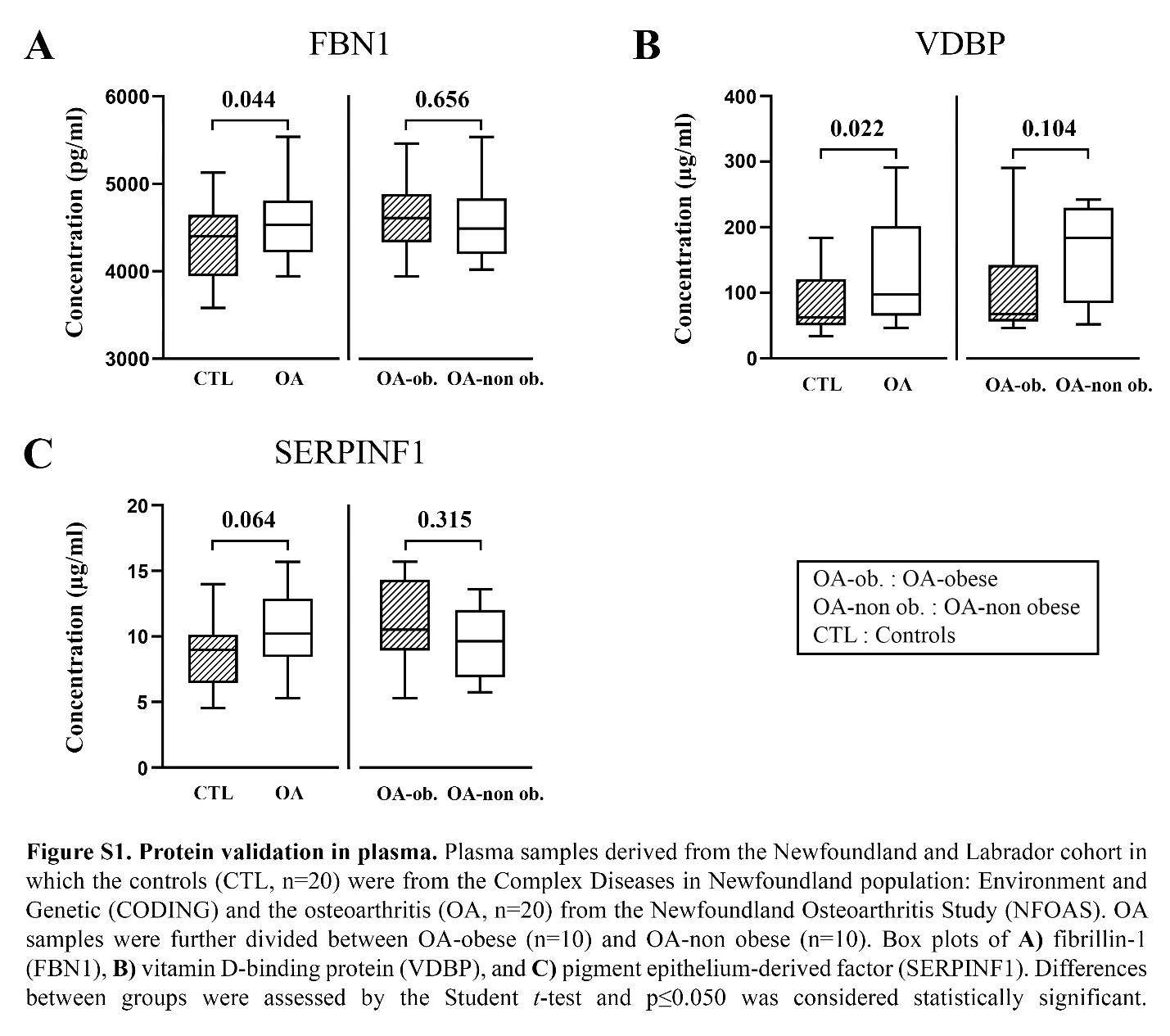

Supplement: Supplementary file 2 — Additional file 2: Figure S1. Protein validation in plasma. [file 13075_2022_2801_MOESM2_ESM.docx]
